# Targeting of non-apoptotic cancer cell death mechanisms by quercetin: Implications in cancer therapy

**DOI:** 10.3389/fphar.2022.1043056

**Published:** 2022-11-16

**Authors:** Hao Yang, Shan Xu, Lidan Tang, Jinhong Gong, Hufeng Fang, Jifu Wei, Dan Su

**Affiliations:** ^1^ Department of Pharmacy, The Affiliated Changzhou No. 2 People’s Hospital of Nanjing Medical University, Changzhou, China; ^2^ Department of Pharmacy, Jiangsu Cancer Hospital, Jiangsu Institute of Cancer Research, The Affiliated Cancer Hospital of Nanjing Medical University, Nanjing, China; ^3^ Department of Clinical Pharmacy, School of Pharmacy, Nanjing Medical University, Nanjing, China

**Keywords:** quercetin, non-apoptotic cancer cell death, autophagy, senescence, mitotic catastrophe

## Abstract

The ultimate goal of cancer treatment is to kill cancer cells, based on the use of various therapeutic agents, such as chemotherapy, radiotherapy, or targeted therapy drugs. Most drugs exert their therapeutic effects on cancer by targeting apoptosis. However, alterations in apoptosis-related molecules and thus assisting cells to evade death, eventually lead to tumor cell resistance to therapeutic drugs. The increased incidence of non-apoptotic cell death modes such as induced autophagy, mitotic catastrophe, senescence, and necrosis is beneficial to overcoming multidrug resistance mediated by apoptosis resistance in tumor cells. Therefore, investigating the function and mechanism of drug-induced non-apoptotic cell death modes has positive implications for the development of new anti-cancer drugs and therapeutic strategies. Phytochemicals show strong potential as an alternative or complementary medicine for alleviating various types of cancer. Quercetin is a flavonoid compound widely found in the daily diet that demonstrates a significant role in inhibiting numerous human cancers. In addition to direct pro-tumor cell apoptosis, both *in vivo* and *in vitro* experiments have shown that quercetin exerts anti-tumor properties by triggering diverse non-apoptotic cell death modes. This review summarized the current status of research on the molecular mechanisms and targets through which quercetin-mediated non-apoptotic mode of cancer cell death, including autophagic cell death, senescence, mitotic catastrophe, ferroptosis, necroptosis, etc.

## 1 Introduction

Even with advances in medical technology and the development of anti-cancer drugs, treatment for most cancers remains a lingering problem. According to GLOBOCAN, approximately 19.3 million patients will be newly diagnosed with cancer and almost 10 million cancer patients died occurred in 2020 worldwide, while the global cancer burden is expected to reach 28.4 million cases in 2040 (Sung et al., 2021). Mainstream cancer therapies include radiotherapy, chemotherapy (anti-cancer drugs), surgery, and new anti-tumor technologies such as immunotherapy and targeted cancer therapy (Furue, 2003; Baskar et al., 2012; Vanneman and Dranoff, 2012). The ultimate goal of all these therapies is to regulate the survival or death of cancer cells, and anti-cancer drugs can kill clonogenic malignant cells by regulating various cell death modes, such as apoptosis, autophagy, senescence, mitotic catastrophe, ferroptosis, necroptosis, etc. (Galluzzi et al., 2018; Sun et al., 2022).

Apoptosis is a programmed cell death (PCD) process that occurs following the stimulation of cells by various death signals, which is characterized by caspase-dependent, cellular contraction, and the formation of apoptotic body (Su et al., 2020). Currently, the majority of chemotherapeutic drugs inhibit the growth of cancer cells by inducing apoptosis, thus providing treatment for various malignancies. However, the inherent apoptotic resistance of cancer cells or the occurrence of a series of pro-survival mutations occurs during the malignant transformation rendering them resistant to apoptosis, which is the main cause of radioresistance and chemoresistance in most cancers (Liu et al., 2010; Carneiro and El-Deiry, 2020; Cao et al., 2021). An early study has demonstrated that the sensitivity of chemotherapeutic drug-induced cell death *via* apoptosis depends on the activation of caspases, such as cytarabine, doxorubicin, and methotrexate, whereas the inactivation of caspases leads to drug resistance in cancer cells (Los et al., 1997). As a key regulator of the mitochondrial apoptotic pathway, overexpression of members of B-cell leukemia/lymphoma-2 (BCL-2) family proteins inhibit apoptosis both in normal cells and tumor cells, which is another drug resistance factor (Kapoor et al., 2020). Overall, most anti-tumor drugs exert their anticancer activity by targeting cancer cells through apoptosis, and apoptosis defective, manifested by mutations, deletions, and/or overexpression of pro-apoptotic genes, contribute to the development of acquired therapy resistance of cancer cells to chemotherapeutic agents. Hence, it is essential to develop adjuvant or alternative drugs that target the non-apoptotic cell death modes.

**FIGURE 1:**
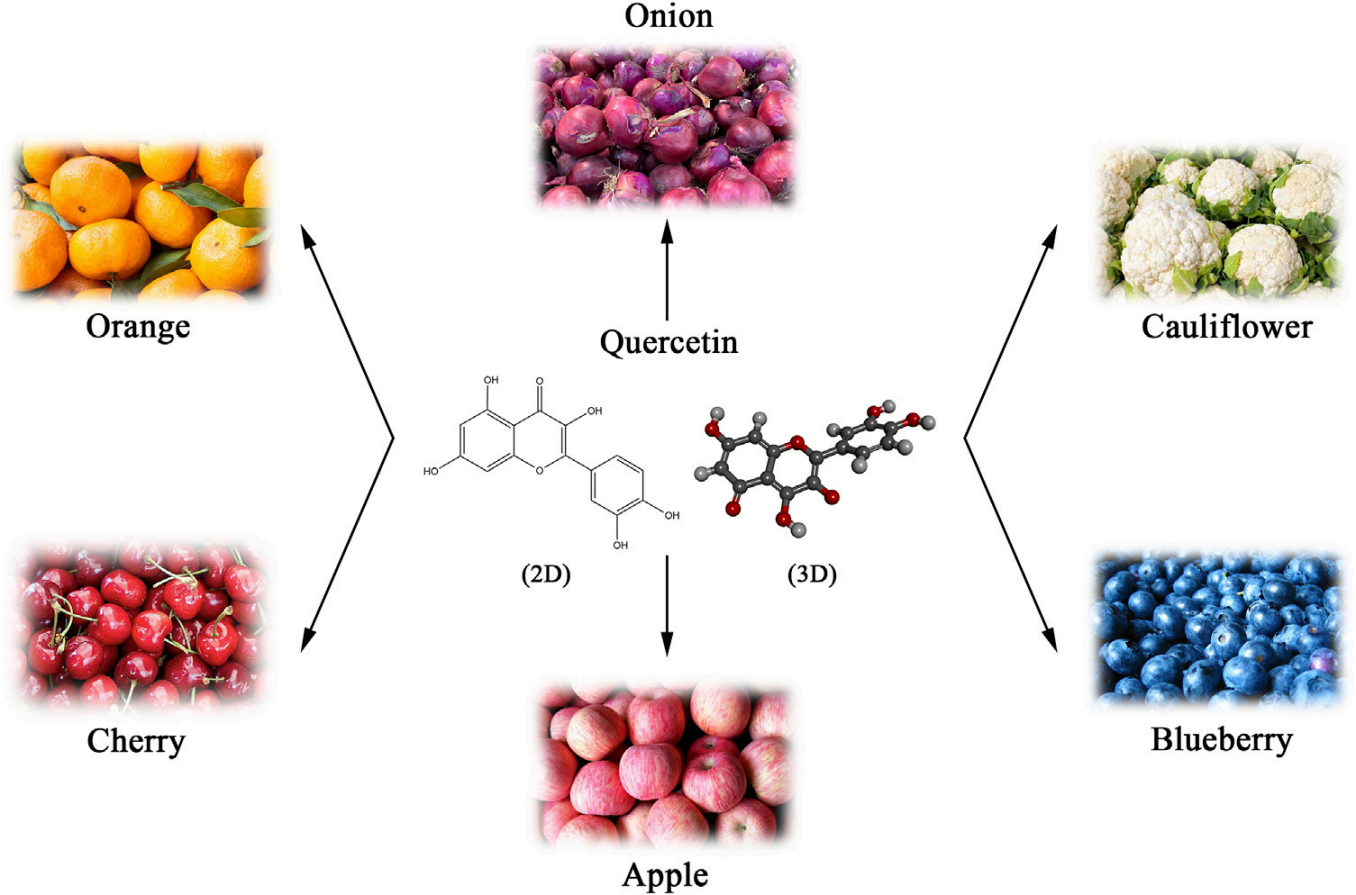
Natural sources and chemical structure (Skeletal formulas and 3D stick representations) of quercetin.

Natural products are an essential source of anticancer lead molecules due to their multi-targeting efficacy and low toxicity, especially flavonoids (Yang H. et al., 2022a; Liao et al., 2022). As a flavonoid with various biological activities, quercetin is abundantly present in plants, fruits, and vegetables, mainly in the form of glycosides, such as onions, apples, blueberries, cauliflower, etc. (Batiha et al., 2020) (Figure 1). Considering its anti-inflammatory and antioxidant abilities as well as the modulating effects on tumor microenvironment, quercetin has been added to functional foods as a dietary supplement for the prevention and/or treatment of diverse diseases such as cancer (Li Y. et al., 2016b; Andres et al., 2018; Reyes-Avendaño et al., 2022). Numerous *in vivo* and *in vitro* research have found that quercetin induces apoptosis in different cancer cell lines and exhibits anti-tumor properties (Hashemzaei et al., 2017; Khorsandi et al., 2017; Safi et al., 2021). Nevertheless, recent studies suggest that quercetin may also kill cancer cells *via* several different mechanisms. The current paper summarizes the literature on the regulation of diverse cancer cell death modes and mechanisms following cancer treatment with quercetin, rather than apoptosis.

## 2 Background information on quercetin

Quercetin, a member of the flavonoid family, with the chemical name of 3,5,7-trihydroxy-2-(3,4-dihydroxy phenyl)-4-Hchromen-4-one (Li Y. et al., 2016b). As a derivative of phenyl benzoyl ketone, quercetin consists of two benzene rings (A and B rings) linked by an oxygenated pyrene ring (C ring), with the flavonoid structure C6 (A ring)-C3 (C ring)-C6 (B ring) as the basic backbone (Figure 1). It is structurally evident that the flavonol skeletal framework of quercetin has five hydroxyl groups located on the 3,3′, 4′, 5 and 7 carbons, therefore it is also known as pentahydroxyflavonol. The relative substitution of various functional groups on the flavonol molecule is the main cause and effect of the wide range of pharmacological activities of quercetin and its metabolites (Khan et al., 2016). For instance, the substitution pattern of the A and B rings of quercetin and the presence and amount of free hydroxyl groups in their backbone are known to be key to the perceived free radical scavenging potential of quercetin (Nabavi et al., 2012). The sugars, lipids, alcohols and a sulphate groups are all able to conjugate to quercetin *via* the O-glycosidic bond, resulting in the formation of its derivatives (Williams and Grayer, 2004). Numerous studies have confirmed that prenylated quercetin analogs show powerful potential in antibacterial properties (Cushnie and Lamb, 2005; Wang et al., 2018). Furthermore, it was found that the natural form of quercetin in the form of a sulphate conjugate had significant anticoagulant activity (Guglielmone et al., 2020). Hence, the investigation and development of functional group substitutions for the different biological effects of quercetin analogues is of considerable and far-reaching importance.

As the most popular dietary flavonoid, quercetin has the wealthiest resources (Figure 1), with β-glucoside being the main form of quercetin that exists in the diet. In the gastrointestinal tract, glycosides are highly dependent on microbially-derived β-glucosidases for hydrolyzing to the aglycon before their absorption and transportation take place (Zhao L. et al., 2022a). In intestinal epithelial cells, quercetin is extensively metabolized to quercetin-3- and quercetin-7-glucuronide. Afterward, they are rapidly metabolized in the liver to methyl, glucuronides, or sulfate conjugates, the forms of quercetin present in circulation (Terao et al., 2011). Notably, the highly lipophilic nature of quercetin determines its relatively low water solubility and bioavailability. Consequently, it is imperative to investigate the absorption, distribution, metabolism, and excretion (ADME) of quercetin, which contributes to understanding its bio-transfer *in vivo*. Yin et al. investigated the pharmacokinetics of quercetin. After oral administration of quercetin (50 mg/kg) to rats (*n* = 5), the pharmacokinetic analysis showed that quercetin peaked after 1 h. The mean plasma concentration (C_Max_) of quercetin was 7.47 ± 2.63 μg/mL, and the average area under the plasma concentration-time curve extrapolated to infinitive time (AUC_0-∞_) and elimination half-life (t_1/2_) was 2,590.5 ± 987.9 mg/L*min and 437.3 ± 54.3 min, respectively (Yin et al., 2019). The distribution of quercetin in the tissues of rats fed with 0.2% quercetin diet for 11 weeks was observed, and its concentration distribution was in the following order: lung > testis > kidney > thymus > heart > liver > brown fat > bone > brain > spleen (de Boer et al., 2005). In addition, researchers exposed pigs to a high-dose quercetin diet [500 mg/kg/day)] for 3 days. The results showed that the distribution of quercetin concentration is ranked by: liver > kidney > brain > heart > spleen. 3-hydroxyphenylacetic acid, benzoic acid, and hippuric acid are the main excretion products of quercetin, the majority of which are excreted through urine and feces, however, when taken in a high amount of quercetin, the lungs are also one of the organs of clearance (Walle et al., 2001; Guo and Bruno, 2015). The safety of quercetin as a dietary supplement also requires consideration. In a double-blind, placebo-controlled crossover trial, the overweight or obese volunteers were administered a relatively low dose of quercetin (150 mg/day) for 6 weeks. The results of blood biochemical tests showed that the parameters of biomarkers of liver and kidney function (alanine transaminase, g-glutamyl-transpeptidase, aspartate transaminase, creatinine, etc.) were within normal limits, indicating no adverse effects of quercetin (Egert et al., 2009). Moreover, continuous administration of quercetin (250–5,000 mg) for 28 days showed no exacerbation of liver enzyme (aspartate transaminase and alanine transaminase) levels in patients with chronic hepatitis C (Lu et al., 2016). However, data related to the safety evaluation of long-term, high-dose quercetin supplementation are still limited. Hence, more in-depth exploration of these issues is needed in future intervention research.

As research progresses, quercetin has received increasing attention for its nutritional and therapeutic potential, benefiting from its wide range of biological activities (Yang et al., 2020). Numerous *in vivo* studies have demonstrated the strong potential of quercetin for anti-diabetes and its complications, together with antioxidant, anti-inflammatory, anti-viral, and significant neurological and cardiovascular-related benefits (Patel et al., 2018; Shi et al., 2019; Yang et al., 2019; Di Petrillo et al., 2022; Zhang et al., 2022). Moreover, growing evidence suggests that in addition to apoptosis, quercetin also exerts anti-tumor effects *via* multiple signaling pathways that induce non-apoptotic cancer cell death modes, making it a promising natural product for the prevention and nutritional management of cancer (Rauf et al., 2018; Tang et al., 2020).

## 3 Therapeutic mechanisms of quercetin targeting non-apoptotic cell death patterns in cancer

Different tumor therapy may induce cancer cell death *via* diverse mechanisms. As a common mode of cell death caused by impaired cytogenetic content, conventional cancer therapy usually evokes cell death by inducing apoptosis, however, the inherent and/or acquired apoptotic resistance of cancer cells persists being a major hindrance to the efficacy of chemotherapy (Farhat et al., 2014; Murray et al., 2014; Fox and Storey, 2015). Chemotherapeutic drugs are also known to kill cancer cells *via* other different mechanisms, including autophagic cell death, mitotic catastrophe, senescence, ferroptosis, necroptosis, etc. (Galluzzi et al., 2018; Sun et al., 2022). Quercetin induces cell death *via* different mechanisms, the most common of which is triggering apoptosis. However, it has been suggested that some other mechanisms are also possible factors for quercetin to trigger cancer-killing (Tang et al., 2020). With the sustained exploration of cell death mechanisms, targeting non-apoptotic cell death modes has emerged as a potentially new mechanism of cell death induced by cancer therapies, which may complement or replace apoptosis-induced cancer cell therapy (Denisenko et al., 2016; Li et al., 2017). This section reviews the various mechanisms of non-apoptotic cell death induced following cancer therapy with quercetin based on the literature conducted over the past 10 years (Tables 1, 2).

**TABLE 1:** Summarized mechanisms of autophagy following treatment with quercetin.

Cells/tumor	Quercetin concentration	Findings and involved mechanisms	References
MG-63 cells Balb/c nude mice	50, 100, and 200 μM 100 mg/kg/day i.g.	Quercetin treatment up-regulated LC3B-II/LC3B-I and down-regulated P62/SQSTM1 expression, suggesting that quercetin increased autophagic flux in MG-63 cells. Specifically, quercetin induced osteosarcoma cell death by inducing excessive autophagy mediated by the ROS-NUPR1 pathway	Wu et al. (2020)
BC3, BCBL1, and BC1 cells	50 nM	Quercetin treatment induced a complete autophagic flux. Furthermore, further accumulation of the lipidated form of the autophagy marker LC3 (LC3-II) was observed in quercetin combined with vesicular proton pump inhibitor-treated BC3, BCBL1, and BC1 cells compared to the single treatments, and the cleavage of PARP in the cells was increased, indicating that the combined treatment increased the cytotoxicity	Granato et al. (2017)
SH-SY5Y Cells	50 nM	Quercetin causes autophagy *via* up-regulation of microtubule-associated protein LC3II bound by autophagic vesicles. In addition, quercetin exerts against Cu-induced toxic damage by regulating the autophagic pathway to restore endoplasmic reticulum homeostasis in cellular organelles	Chakraborty et al. (2022)
MIA Paca-2^GEMR^ cells	25, 50, 100, and 200 μM	Quercetin treatment of MIA Paca-2 GEMR cells for 24 h or 48 h resulted in a decrease in RAGE protein expression levels and a dose-effect increase in the percentage of autophagic cells	Lan et al. (2019)
LM3 cells BALB/c nude mice	80 and 120 μM 100 mg/kg/day i.g.	Quercetin treatment induced cellular autophagy by upregulating LC3 expression and downregulating P62 expression in a time-dependent manner. These effects partially depended on quercetin downregulation of JAK2 and STAT3 activation	Wu et al. (2019)
A549 cells	20, 40, and 80 μM	Quercetin significantly enhanced TNF-related apoptosis-inducing ligand (TRAIL)mediated lung cancer cell death by activating autophagic flux.	Moon et al. (2015)
U251 and U87 cells	30 μM	Quercetin blocked t-AUCB-induced autophagy in a human glioblastoma cell line by inhibiting the expression of Hsp27 and Atg7	Li et al. (2016)
AGS and MKN28 cells	40 μM for AGS cells 150μM for MKN28 cells	Quercetin-induced autophagy decreased its therapeutic effect in gastric cancer cells. miR-143 targeting GABARAPL1 effectively inhibited autophagy in gastric cancer cell lines, which could improve the efficacy of quercetin.	Du et al. (2015)
CAOV3 and primary ovarian cell	10, 20, and 40 μM for CAOV3 cells 20, 40, and 80 μM for primary ovarian cell	Quercetin treatment triggers protective autophagy through activation of the p-STAT3/Bcl-2 axis induced by endoplasmic reticulum stress.	Liu et al. (2017a)

**TABLE 2:** Summarized mechanisms of non-apoptotic death of cancer cells following treatment with quercetin.

Cells/tumor	Quercetin concentration	Cell death type	Findings and involved mechanisms	References
T24 cellS	40, 60, or 80 μM	Senescence	Quercetin treatment showed an increase in the percentage of the nuclear characteristic of the senescence process, the cell nucleus area, as a result of the morphological analysis of the cell nuclei	Adami et al. (2021)
Colo-320 and Colo-741 cells	25 µM	Senescence	After treatment with quercetin, Lamin B1, p16, and cyclin B1 immunoreactivity were increased in Colo-320 and Colo-741 cells, which is usually considered a marker of cellular senescence	Özsoy et al. (2020)
C6 and U87 cells	25 μM	Senescence	Treatment with quercetin for four consecutive days increased the levels of senescence markers in C6 and U87 cells, furthermore senescence-associated cell morphological changes such as flattening, increased particle size, and cell enlargement could be observed. In addition, HDAC inhibited the positive effects of quercetin-induced senescence	Vargas et al. (2014)
U87-MG, U251 and SHG44 cells	50,100, or 200 μM	Senescence	Quercetin promotes glioma cell senescence *via* inhibition of the Ras/MAPK/ERK signaling pathway in a dose-dependent manner	Pan et al. (2015)
HeLa cells	30, 60, or 90 μM	Senescence	After 18 h of quercetin treatment, Hela cell density increased in the G2/M phase of the cell cycle, reflecting cell cycle arrest at that stage	Bishayee et al. (2013)
U251 cells	10, 20, 30, or 40 μM	Senescence	The number of U251 glioblastoma cells in sub-G2/M phase increased after treatment with quercetin (10–30 μM) for 24 h, indicating that quercetin caused G2/M phase arrest	Liu et al. (2017b)
SKOV3 and U2OSPt cells	10 and 50 µM	Senescence	After treatment with quercetin in SKOV3 and U2OSPt cells, cell cycle distribution was significantly altered. Quercetin treatment affected the cell cycle in G1/S and G2/M phases by decreasing cyclin D1 and cyclin B1 levels	Catanzaro et al. (2015)
A549 cells	10, 30, or 60 μM	Mitotic catastrophe	Quercetin treatment exerted the inhibitory effect on the proliferation of A549 cells mainly *via* the induction of mitotic catastrophe and apoptosis. The mechanism may involve the perturbation of mitotic microtubules, leading to the monopolar spindle formation, which leads to the failure of cytokinesis	(Klimaszewska-Wiśniewska et al., 2017)
Hepa1c1c7 cells	0.01 μM	Mitotic catastrophe	Low concentrations of quercetin treatment produced mitotic catastrophe. Disproportionate DNA segregation was observed when quercetin concentration was as low as 0.01 μM	Jackson et al. (2016)
MCF-7 and MDA-MB-231 cells	0.1, 1, and 10 μM	Ferroptosis	Quercetin treatment upregulated intracellular iron, carbonyl protein, and MDA levels in breast cancer cells in a dose-dependent manner. The pharmacological effects of quercetin on killing breast cancer cells might be related to the promotion of TFEB expression and nuclear transcription, which induce the occurrence of iron death	An and Hu, (2022)
HepG2, Hep3B, MDA-MB- 231, and HCT116 cells	50 μM	Ferroptosis	Quercetin possesses the effect of promoting lysosome-dependent ferritin degradation and free iron release, which in synergy with quercetin-induced ROS generation leads to lipid peroxidation and ferroptosis.	Wang et al. (2021a)
MCF-7 cells	50 μM	Necroptosis	Quercetin significantly inhibited MCF-7 cell viability and proliferation *via* activation of apoptotic and necroptosis signaling pathways. Quercetin possesses a necroptosis-inducing effect possibly by increasing the expression of RIPK1 and RIPK3	Khorsandi et al. (2017)
Giant cell tumor of bone	120 μM	Necroptosis and autophagy	The ultrastructural changes observed in giant cell tumors of bone cultured quercetin for 24 h corresponded mainly to necroptosis, secondary necrosis, and autophagocytosis	Estrada-Villaseñor et al. (2021)
4T1 cells nude mice with subcutaneous injection of 4T1 cells (10^7^/mL)	Not mentioned (*in vivo*) 20 mg/kg/day, i.p. (*in vivo*)	Pyroptosis	Quercetin-treated BCRD rat, serum IFN-γ, IL-10, and IL- 2 levels were significantly upregulated, which probably *via* promoting anti-tumor immune response. In addition, quercetin partially reversed the pyroptosis on LPS-cultured 4T1 cells *in vitro*, as evidenced markedly by upregulating the ASC, NLRP3 and Caspase-1	Zhu et al. (2022)

### 3.1 Quercetin and autophagy

Autophagy is a type II programmed cell death that prevents tumor initiation and suppresses cancer progression in early tumorigenesis. As a key regulator of cellular metabolism during starvation, autophagy normally protects cells from stressors like nutrient deprivation. (Kimmelman and White, 2017). Stimulation of the tumor microenvironment usually triggers autophagy, such as nutrient deprivation, reactive oxygen species (ROS), hypoxia, and pathogen invasion (Su et al., 2015). Typically, autophagy passes through distinct stages including induction of autophagy, nucleation of the autophagosome, expansion, and elongation of autophagosomal membranes, closure, and fusion of autophagosomes with lysosomal membranes, and degradation and recirculation of intracapsular products (Li et al., 2020). During cancer cell survival, autophagy plays dichotomous role, exerting dynamic tumor-suppressive or tumor-promoting effects at different stages or settings (White, 2015; Levy et al., 2017). Although autophagy promotes tumorigenesis, abundant evidence indicates that the triggering of autophagy may limit tumor progression and improve response to cancer therapy (White, 2015; Amaravadi et al., 2019). In addition to the cytoprotective and cytotoxic forms of autophagy, Gewirtz DA proposed in a review published in 2014 that autophagy is actually populated by at least two additional players, a nonprotective form of autophagy and a cytostatic form of autophagy (Gewirtz, 2014). As a survival response, cytoprotective autophagy enables tumor cells to evade apoptotic signals and become resistant to chemotherapy and radiotherapy. When cytoprotective autophagy is blocked, it can enhance the sensitivity of tumor cells to chemotherapy and increase apoptosis in cancer cells (Ulasov et al., 2020; Xu et al., 2022). For this reason, multiple clinical trials are currently being conducted for treating cancer by targeting cytoprotective forms of autophagy using autophagy inhibitors (e.g., chloroquine or hydroxychloroquine) in combination with various conventional therapeutic modalities tto achieve improved efficacy (Gewirtz, 2014). Although autophagy promotes tumorigenesis, abundant evidence indicates that the triggering of autophagy may limit tumor progression and improve response to cancer therapy (White, 2015; Amaravadi et al., 2019). Cytotoxic autophagy promotes tumor cell death, either by killing its own cells or by acting as a precursor to apoptosis (Sui et al., 2013; Gewirtz, 2014). Generally, the key to distinguish between cytotoxic autophagy and cytoprotective autophagy is to observe the sensitivity of tumor cells to the therapeutic modality. Besides, Thorburn A and Gewirtz DA observed induction of another form of autophagy by radiation, which would be termed ‘‘nonprotective’’, whose inhibition is neither affect cell proliferation nor apoptosis (Bristol et al., 2013). In a particular tumor cell line, the manner of treatment may determine whether autophagy is cytoprotective or nonprotective (Gewirtz, 2016). In 2014, Gewirtz DA proposed for the first time a novel form of autophagy, namely cytostatic autophagy (Sharma et al., 2014). This study noted that combined treatment with vitamin D (or a vitamin D analogue, EB 1089) and radiation resulted in more pronounced growth inhibition in non-small cell lung cancer cells than radiation alone, as well as greater sensitivity to radiation. In addition, they identified that radiation-induced conversion of cytoprotective autophagy to cytostatic autophagy. Considering that cytostatic autophagy usually mediates cell growth inhibition, this form of autophagy may affect the effectiveness of chemotherapy and/or radiotherapy targeting tumor cell growth arrest. Unfortunately, there is no well-defined biochemical or molecular signature that distinguishes these forms from each other.

**FIGURE 2:**
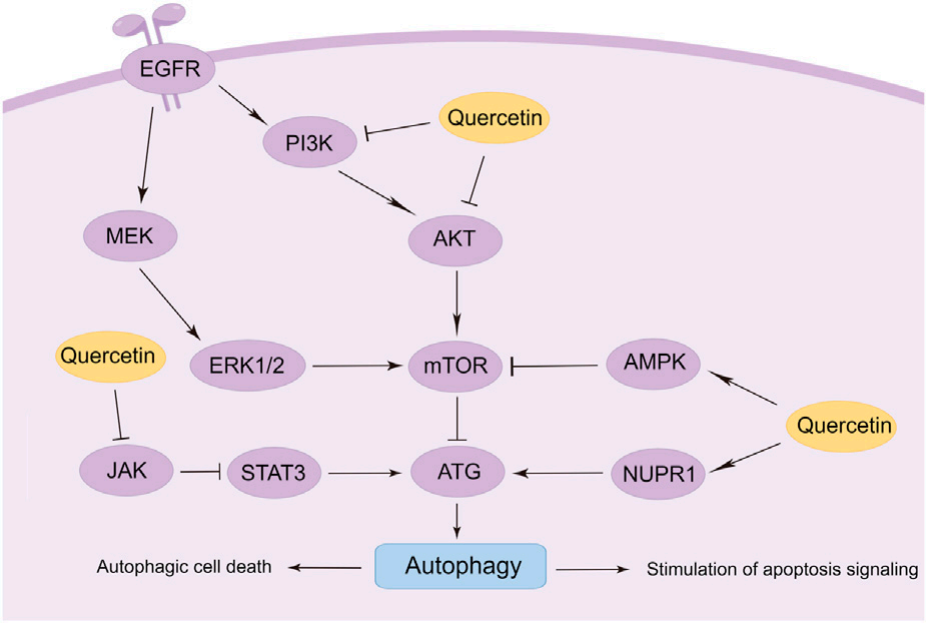
Mechanisms of autophagy modulation by quercetin in cancer (By Figdraw).

Quercetin has been demonstrated to promote and control the regulation of autophagy in different types of cancers. Treatment of Burkitt lymphoma cell lines with quercetin, LC3Ⅰ was converted to LC3Ⅱ, which is commonly considered a biomarker of autophagy, suggesting that quercetin induced a complete autophagic flux (Granato et al., 2016). The same findings were obtained in HL-60 xenograft mice, where quercetin administration induced the conversion of LC3-I to LC3-II and activated autophagy proteins, indicating that quercetin treatment triggered the autophagic process and thus anti-tumor growth (Calgarotto et al., 2018). In addition to upregulating the LC3-II/I ratio in a dose-dependent manner, a large number of double membranes were observed in quercetin-treated glioma cell lines (Bi et al., 2016). In SH-SY5Y cells, quercetin also induced autophagy by upregulating LC3II. This study also revealed that quercetin restored organelle endoplasmic reticulum homeostasis and alleviated the cytotoxic damage induced to Cu by regulating the autophagic pathway (Chakraborty et al., 2022). Moreover, the highest number of cellular autophagic vacuoles was observed in HeLa cells treated with 50 μM quercetin, but the number decreased instead at higher doses (Wang et al., 2016). In lung cancer cells, quercetin treatment also significantly increased Beclin 1 protein expression and the number of autophagic vacuoles and autophagosomes (Guo et al., 2021). Contrasting results were observed in human glioblastoma cell lines, where quercetin treatment increased the formation of autophagic lysosomal vesicles but had no effect on the expression of Beclin-1 (Kim et al., 2013). Mammalian target of rapamycin protein (mTOR) is one of the key proteins regulating the autophagic process, causing phosphorylation and inactivation of autophagy protein (ATG), thus inhibition of mTOR leads to upregulation of ATG and initiation of the autophagic process (Kim et al., 2011; Kim and Guan, 2015). In an *in vivo* and *in vitro* study, quercetin was used to treat breast cancer. This study indicated that quercetin induced cellular autophagy by inactivating the protein kinase B (Akt)-mTOR pathway, while the use of Akt-mTOR pathway inducers and autophagy inhibitors further confirmed the involvement of the Akt-mTOR pathway in quercetin-induced autophagy (Jia et al., 2018). Similarly, autophagosomes and autophagolysosomes were significantly increased in hepatocellular carcinoma (HCC) cells after quercetin treatment. By using pathway-specific inhibitors or activators, it is suggested that quercetin stimulates autophagy by inactivating the AKT/mTOR pathway and activating the MAPK pathway (Ji et al., 2019). In addition, quercetin significantly induced cellular autophagy and enhanced gemcitabine-induced cytotoxicity by inhibiting the phosphatidylinositol-3-kinase (PI3K)/AKT/mTOR axis in pancreatic cancer cells (Lan et al., 2019). In addition, quercetin may also induce autophagy in cancer cells through other mechanisms. Wu et al. found that quercetin treatment induced cellular autophagy by upregulating LC3 expression and downregulating p62 expression in a time-dependent manner. These effects were at least partially dependent on quercetin downregulation of Janus kinase 2 (JAK2) and signal transducer and activator of transcription 3 (STAT3) activation (Wu et al., 2019). In primary ovarian cancer (OC) cells, quercetin treatment activates the p-STAT3/Bcl-2 axis followed by induction of protective autophagy (Liu, Gong, et al., 2017). In the study of quercetin-induced osteosarcoma cell death, quercetin promoted the expression of autophagy-related genes through activation of NUPR1 gene activity, which subsequently triggered excessive autophagy in cancer cells (Wu et al., 2020). There are complex interactions between autophagy and apoptosis. A study confirmed that quercetin significantly enhanced tumor necrosis factor (TNF)-related apoptosis-induced ligand-mediated lung cancer cell death through activation of autophagy (Moon et al., 2015) (Figure 2).

Interestingly, the combination of quercetin with other drugs can improve the antitumor efficacy of quercetin by inhibiting or inducing autophagy. Granat et al. demonstrated that quercetin treatment induced a complete autophagic flux in the primary effusion lymphoma (PEL) cell lines. Furthermore, further accumulation of the lipidated form of the autophagy marker LC3 (LC3-II) was observed in quercetin combined with vesicular proton pump inhibitor-treated BC3, BCBL1, and BC1 cells compared to the single treatments (Granato et al., 2017). Another study demonstrated that quercetin-induced autophagy reduced its therapeutic effect on gastric cancer (GC) cells, and treatment with quercetin combined with miR-143 agonist, an inhibitor of autophagy in GC cells targeting GABARAPL1, could improve the antitumor efficacy of quercetin (Du et al., 2015). In addition, quercetin combined with soluble epoxide hydrolase inhibitor (t-AUCB) promotes cell death by inducing autophagy blockade, which may be a potential strategy for the treatment of glioblastoma (Li J. et al., 2016a).

### 3.2 Quercetin and senescence

Cellular senescence, a permanent state of cell cycle arrest due to various cancer-induced stresses, inhibits cancer by irreversibly preventing cell proliferation and is one of the protective mechanisms against cancer in addition to apoptosis (Collado et al., 2007; Calcinotto et al., 2019). Cellular stress, DNA damage, and oncogene activation are among the stimuli that cause cellular senescence (Ma et al., 2018; Jochems et al., 2021). Besides the cell cycle arrest, the morphological features that accompany cellular senescence include cellular enlargement, flattening, vacuolization, and occasionally multinucleation or increased nuclear occupancy. However, these changes are usually only observed during cellular senescence *in vitro* cultures, while *in vivo* senescent cells maintain normal morphologically determined tissue structure (Collado and Serrano, 2006; Muñoz-Espín and Serrano, 2014; Bernadotte et al., 2016). In cultured cells and/or tissues, the detection of a collection of biomarkers is used to define senescence. The histochemical assay for β-galactosidase activity is the most widely used assay for senescence. Common mediators of senescence, including p16, ARF, p53, p21, p15, and p27, are also typical biomarkers of senescence (Muñoz-Espín and Serrano, 2014). In addition, alterations in Lamin B1 levels are a common feature of many types of senescence (Lukášová et al., 2018; Radspieler et al., 2019). Generally, senescence is considered as an effective anti-tumor mechanism through which cancer cells proliferate and inhibit malignant progression. Furthermore, senescence is one of the physiological tumor suppressor mechanisms that limit the progression of tumors from benign tumor lesions to malignant ones. As a consequence of these effects, it has cancer suppressive potential, while senescence-associated secretory phenotype (SASP) plays an important role in the pathophysiological role of senescent cells (Wyld et al., 2020; Özsoy Gökbilen et al., 2022). However, the signaling pathway of senescence is also a key effector of radiotherapy and chemotherapy injury, which may lead to reduced recovery in patients receiving anticancer therapy and may result in cancer recurrence. On the other hand, growing evidence indicates that senescent cells may induce proliferative pathology in cancer, moreover, SASP factors may also trigger epithelial-mesenchymal transition (EMT) in premalignant epithelial cells (Liu and Hornsby, 2007; Kuilman et al., 2008). Therefore, the use of senolytic agents to remove senescent cells may play a key role in preventing cancer recurrence.

It was found that quercetin can induce senescence in cancer cells. In quercetin-treated T24 bladder cancer cells, an increase in the percentage of nuclei during cellular senescence was observed by nuclear morphometric analysis (NMA) (Adami et al., 2021). Furthermore, the immunoreactivity of Lamin B1, p16, and cyclin B1, markers of cellular senescence, was also increased in Colo-320 and Colo-741 cells after treatment with quercetin (Özsoy et al., 2020). Pan et al. found that the mechanism by which quercetin promotes cellular senescence may be *via* inhibition of the Ras/MAPK/ERK signaling pathway in a dose-dependent manner (Pan et al., 2015). In another study, treating C6 and U87 cells with quercetin for 4 consecutive days elevated levels of cellular senescence markers, furthermore senescence-associated cell morphological changes such as flattening, increased particle size, and cell enlargement could be observed. The researchers also found that histone deacetylase (HDAC) played a key role in quercetin-induced senescence. HDAC inhibitors significantly enhanced quercetin-induced senescence in human and rat glioma cell lines (Vargas et al., 2014). Quercetin triggers cellular senescence probably by increasing the expression of tumor suppressor gene p53 and cell cycle protein-dependent kinase (CDK) and cyclin B1 inhibitors p21, p27, thus inducing cell cycle arrest in G1 and G2/M phases (Tang et al., 2017; Özsoy Gökbilen et al., 2022). A study in 2013 showed that quercetin treatment for 18 h increased Hela cell density in the G2/M phase of the cell cycle, reflecting cell cycle arrest at this stage (Bishayee et al., 2013). Moreover, Liu et al. confirmed the same finding in different cell lines. After quercetin treatment for 24 h, the number of U251 glioblastoma cells in the sub-G2/M phase increased, indicating that quercetin induced G2/M phase arrest and thus inhibited U251 cell proliferation (Liu, Tang, et al., 2017). Cantanzaro et al. designed a study to investigate the factors by which quercetin affects the G1/S and G2/M cell cycles. The results showed that quercetin treatment on SKOV3 and U2OSPt cells for 48 h significantly altered the cell cycle distribution, possibly affecting the G1/S and G2/M phases of the cell cycle by decreasing the levels of cyclin D1 and cyclin B1 (Catanzaro et al., 2015).

**FIGURE 3:**
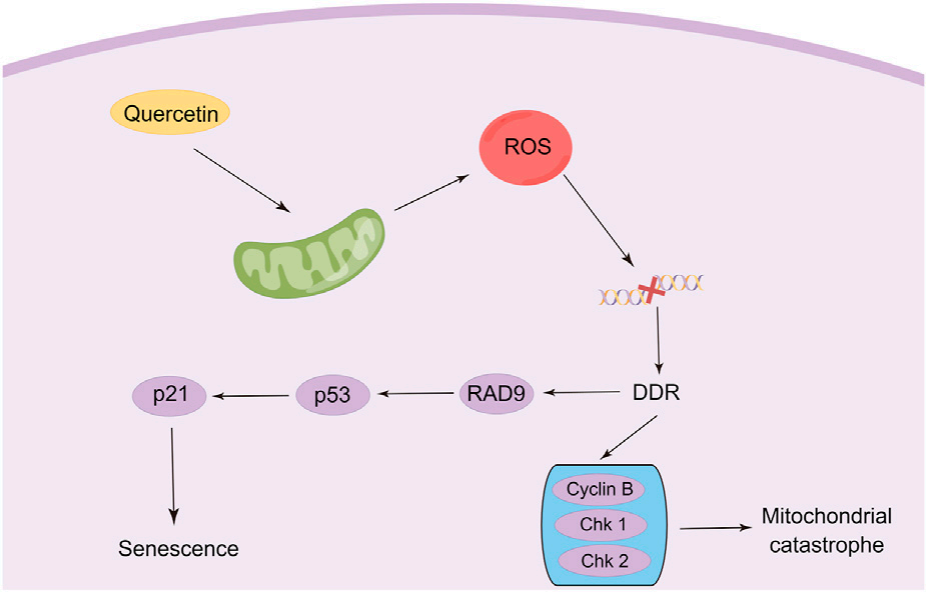
Modulation of senescence and mitotic catastrophe in cancer cells by quercetin (By Figdraw).

Quercetin is usually not as powerful as targeted senolytics agents, but quercetin in combination with other senolytic agents can be more effective in removing senescent cells. Zhu Y et al. first reported a senolytic cocktail (Dasatinib combined with quercetin) that effectively killed senescent cells with the help of small interfering RNA (Zhu et al., 2015). Subsequently, in two open-label Phase I pilot studies, researchers demonstrated for the first time that senolytic agent (Dasatinib combined with quercetin) significantly reduces human senescent cell burden and provided preliminary evidence that senolytic agents may alleviate physical dysfunction in patients with idiopathic pulmonary fibrosis (IPF) (Hickson et al., 2019; Justice et al., 2019). Notably, some chemotherapeutic agents that exert their antitumor effects *via* the induction of cancer cell senescence also trigger cellular senescence in normal cells, which drives the development of a malignant phenotype in residual living tumor cells (Demaria et al., 2017; Bruni et al., 2019). Quercetin has been demonstrated to reduce chemotherapeutic drug-induced aging in combination with anticancer drugs. Recent studies have established that quercetin pre-treatment can prevent doxorubicin-induced senescence in normal cells by reducing the number of senescent cells and the production of SASP factors (Bientinesi et al., 2022). Furthermore, quercetin pre-treatment may also protect normal cells from doxorubicin treatment-induced ROS damage, by increasing cellular antioxidant defense. In another study, a quercetin derivative (quercetin-3-*O*-β-D-glucuronide) also showed a good inhibitory effect on cellular senescence in doxorubicin-treated HDFs and HUVECs cells (Yang et al., 2014). Considering that cancer cells may use senescence as an escape strategy from cancer treatment, the use of quercetin may selectively remove spontaneous cancer cells previously induced by chemotherapy and/or radiotherapy. Therefore, the use of some phytochemicals as senolytic agents or protectors, such as quercetin, is probably useful for overcoming tumor resistance (Figure 3).

### 3.3 Quercetin and mitotic catastrophe

Mitotic catastrophe is another critical non-apoptotic mechanism of cancer cells, defined by the Nomenclature Committee on Cell Death (NCCD) in 2012, which usually occurs after massive damage to DNA following ROS generation (Galluzzi et al., 2012). Cells undergoing mitotic catastrophe are often in partnership with other mechanisms of cell death, such as autophagy, senescence, or, necroptosis (Eom et al., 2005; Zhao W. et al., 2022b; Egorshina et al., 2022). Recent studies have found that the therapeutic efficiency of anticancer modalities such as radiotherapy and chemotherapy can be reinforced by stimulating mitotic catastrophe, which is also a promising way to overcome multidrug resistance (Pérès et al., 2015; Bai et al., 2017). In addition, ionizing radiation (IR) can also trigger immune cell mitotic catastrophe (Adjemian et al., 2020). Hence, the stimulation of mitotic catastrophe constitutes a new direction for tumor therapy. However, very few studies have evaluated the effects of quercetin on mitotic mutations, and the available studies have only found that quercetin induced mitotic mutations, but precisely little has been done to characterize exactly what signals are involved. Jackson et al. characterized the effect of different concentrations of quercetin on mitotic mutagenesis in Hepa1c1c7 cells. The results demonstrated that aberrant mitotic images were observed at concentrations as low as 0.01 μM quercetin, showing disproportionate DNA segregation (Jackson et al., 2016). Sufficient evidence documented that such mitotic abnormalities may eventually lead to mitotic catastrophes (Zhu et al., 2005). Thus, it appears that utilizing lower doses of quercetin to trigger mitotic catastrophes would significantly limit the side effects of the administration of large doses of quercetin anti-tumor. In another study, to investigate whether quercetin can induce growth inhibition in A549 cells by altering the cell cycle, image cytometric analysis of cellular DNA content was used to assess the effect of quercetin on cell cycle distribution. It was found that quercetin treatment dose-dependently resulted in a decrease in cell cycle distribution in G_0_/G_1_-phase accompanied by an increase in cell cycle distribution in G_2_/M-phase in A549 cells (Kobayashi et al., 2008). Moreover, the researchers attribute the failure of cytokinesis to quercetin-induced mitotic catastrophe, with monopolar spindle formation caused by perturbation of mitotic microtubules as a possible mechanism. However, the mechanism of quercetin in regulating mitotic catastrophe needs further elucidation (Figure 3).

### 3.4 Quercetin and ferroptosis

Ferroptosis, a novel mode of cell death first introduced in 2012, depends on intracellular iron and differ from apoptosis, necroptosis, and autophagy in morphology and biochemistry (Dixon et al., 2012; Lei et al., 2022). Ferroptosis is mainly characterized by iron ion accumulation and ROS-induced lipid peroxidation, which morphologically induces marked mitochondrial contraction, increased membrane density, and reduction or disappearance of mitochondrial cristae (Xie et al., 2016; Yang and Stockwell, 2016). The important role of ferroptosis in oxidative stress, iron metabolism, inflammation, and amino acid metabolism has been convincingly established (Galaris et al., 2019; Sun et al., 2020; Yang J. et al., 2022b). Correspondingly, ferroptosis involves multiple physiological and pathological processes, including hematological disorders, ischemia-reperfusion injury, renal injury, and particularly tumor inhibition (Mou et al., 2019; Chen et al., 2021; Li et al., 2021; Zhao et al., 2021).

**FIGURE 4:**
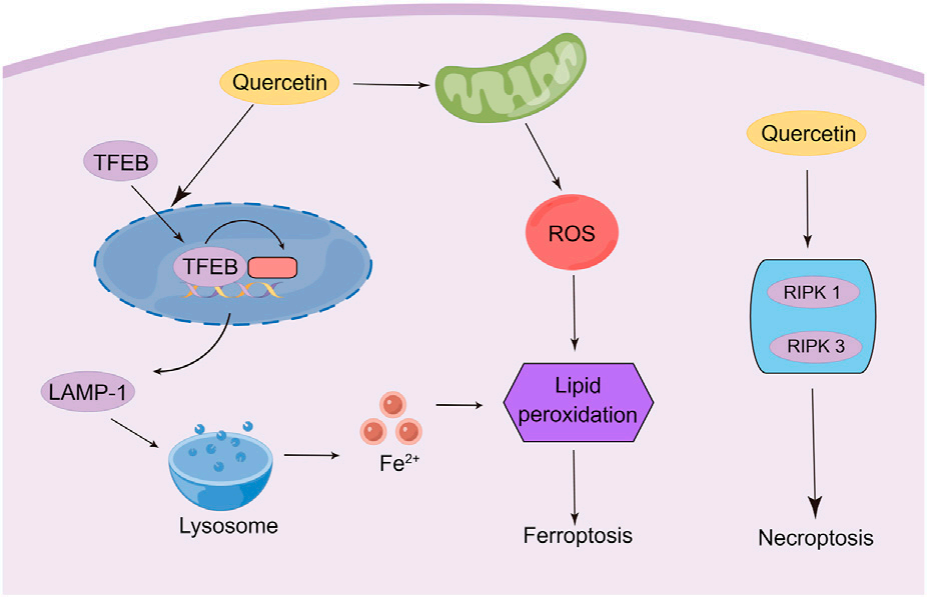
Modulation of ferroptosis and necroptosis in cancer cells by quercetin (By Figdraw).

Recently, it has been found that quercetin exerts anti-tumor effects *via* triggering ferroptosis to induce cancer cell death. The activation of ferroptosis involves multiple signaling pathways. Recent research has identified a new ferroptosis-inducing pathway stimulated by autophagy, namely autophagy-dependent ferroptosis, which is selective autophagy that contributes to ferroptosis (Pierzynowska et al., 2021). During autophagy, the macromolecules in need of degradation are engulfed by phagophores, followed by acid hydrolase digestion of their contents by the lysosome. Transcription factor EB (TFEB) is the master gene for lysosomal biogenesis and autophagy (Settembre et al., 2011; Medina et al., 2015). These findings enlighten us that lysosomal storage diseases (LSD) and related molecular pathogenesis may involve the regulation of ferroptosis. Wang et al. found that the induction of cell death by quercetin could be reversed by lysosomal inhibitors and knockdown of the TFEB, which indicated the involvement of lysosomes in quercetin-induced cell death (Wang Z. X. et al., 2021b). In addition, quercetin promotes ferritin degradation, free iron release, and lipid peroxidation, which was induced by promoting nuclear TFEB and transcriptional activation of lysosomal genes that induce lysosomal activation and inducing ROS production. The synergistic effect of both together leads to iron death. Interestingly, another research also demonstrated that TFEB-mediated lysosomal activation plays an important role in quercetin-induced ferroptosis (An and Hu, 2022). In breast cancer cells, quercetin treatment induced the onset of ferroptosis by promoting TFEB expression and nuclear transcription. Further mechanistic studies showed that the degradation of ferritin and release of ferric ions were regulated by the lysosome-related gene LAMP-1, which was up-regulated due to the high expression of TFEB in the nucleus. Notably, quercetin exhibits a therapeutic effect in several non-tumor disease models due to its significant antioxidant activity characterized by reduced malondialdehyde (MDA) and lipid ROS levels and increased glutathione (GSH) levels, which contradicts the induction of ferroptosis in tumor cells (Wang Y. et al., 2021a; Jiang et al., 2022). ROS is a double-edged sword, and the heterogeneity of tumor cells and non-tumor cells leads to their different responses to ROS. Therefore, quercetin may exhibit opposite abilities in regulating ROS in different cells. Thus, the mechanisms and signaling pathways of quercetin regulation of ferroptosis for cancer treatment still need to be further elucidated (Figure 4).

### 3.5 Quercetin and necroptosis

As another important mechanism of cancer cell death, necroptosis was initially found to be an alternative to apoptosis following the involvement of death domain receptors (Degterev et al., 2005). Triggering necrosis in cancer cells is a promising way to avoid the failure of cancer chemotherapy due to apoptosis resistance by bypassing the apoptotic pathway to induce cancer cell death. As such, the molecular mechanisms of necroptosis have been well investigated, which depends critically on receptor-interacting serine-threonine kinase 1 (RIPK1), RIPK3, and mixed lineage kinase domain-like (MLKL), regardless of the upstream trigger (Hu et al., 2022; Yan et al., 2022). Currently, only limited experiments have shown the induction of necroptosis in cancer cells by quercetin. A study was conducted to observe the ultrastructural changes of quercetin on giant cell tumor of bone (GCTB) cells. The researchers demonstrated that, in addition to autophagy, quercetin treatment affected all histological components of necroptosis and secondary necroptosis by increasing the expression of RIPK1 (Estrada-Villaseñor et al., 2021). Another study reported that quercetin treatment of MCF-7 breast cancer cells depended on multiple cell death pathways, which mainly involve necroptosis (Khorsandi et al., 2017). This study indicated that quercetin induced necroptosis mainly through increasing the expression of RIPK1 and RIPK3. However, other studies indicated that quercetin treatment inhibits M1 macrophage/microglia polarization after spinal cord injury by inhibiting signal transducer and activator of transcription-1 (STAT1) and nuclear factor kappa-B (NF-κB) pathways, which ultimately results in partial alleviation of necrosis of Oligodendrocytes (Fan et al., 2019). The contradictory findings suggest that quercetin may possess the ability to specifically identify and kill tumor cells, which results in opposed effects in tumor cells and non-tumor cells. However, numerous types of research are still needed to confirm this conjecture (Figure 4).

### 3.6 Quercetin and pyroptosis

Pyroptosis, programmed cell death in the form of inflammation, is mediated by the gasdermin family (GSDMs) (Kovacs and Miao, 2017). In the canonical pathway of pyroptosis, certain inflammasomes drive cysteinyl aspartate specific proteinase-1 (Caspase-1) activation, leading to cleavage of gasdermin D (GSDMD) and activation of the inactive cytokines like interleukin-18 (IL-18) and interleukin-1beta (IL-1β), ultimately triggering pyroptosis (He et al., 2015; Schneider et al., 2017). Recent evidence suggests that pyroptosis induces a strong inflammatory response and shows a strong tumor regression effect (Fang et al., 2020). Currently, quercetin has not been reported to possess anti-tumor effects through the regulation of pyroptosis. However, it has been investigated that the specific role of quercetin in breast cancer-related depression (BCRD), in which the inhibition of pyroptosis and promotion of immune response are the main mechanisms for effectively mitigating the progression of BCRD (Zhu et al., 2022). In this study, quercetin partially reversed the pyroptosis on LPS-cultured 4T1 cells *in vitro*, as evidenced markedly by upregulating the card structural domain, NLR family pyrin structural domain (NLRP3), and Caspase-1. In addition, quercetin also promoted an anti-tumor immune response in xenograft mice. Quercetin treatment significantly upregulated the levels of interferon-gamma (IFN-γ), interleukin-10 (IL-10), and interleukin-2 (IL-2) in BCRD Mice. However, the modulation pathways of pyroptosis, especially the immunological effects, remain to be further elucidated after the treatment of cancer with quercetin.

## 4 Summary and future perspective

In summary, this paper reviews the progress of pharmacological research on the non-apoptotic cell death modes induced by quercetin in cancer cells in the last decade. Various cancer cell death modes are interrelated, and regulators of different death modes may crosstalk each other, causing shifts between modes and even accelerating or alleviating tumor cell death. The crosstalk between these cell death mechanisms is complex. For example, apoptosis, autophagy and necrosis are interrelated. Autophagy may promote or antagonize apoptosis through multiple mechanisms, as many regulators, including the mTOR kinase pathway, Beclin 1, caspases, and p53, are involved in both autophagy and apoptosis (Nikoletopoulou et al., 2013; Gali-Muhtasib et al., 2015). Furthermore, a genetic relationship exists between autophagy and aging. On the one hand, the aging program controls the activation of autophagy, on the other hand, different types of autophagy regulation can act through either an anti- or pro-senescence mechanism (Kang and Elledge, 2016). Most anti-cancer drugs exert their efficacy in killing cancer cells mainly by inducing apoptosis, however, apoptosis-resistant cancer cells are often present in the advanced stages of tumor formation and metastasis. Fortunately, some plant-derived components may induce the death of cancer cells resistant to apoptotic stimuli through other non-apoptotic mechanisms. Emerging evidence indicates that quercetin indirectly kills cancer cells by promoting the modes of apoptosis, mitotic catastrophe, senescence, ferroptosis, necroptosis, and pyroptosis. Correspondingly, the induction of each non-apoptotic cancer cell death mode by quercetin is dependent on the up- or down-regulation of some survival-related mediators, such as mTOR, AKT, p53, p21, p15, p27, RIPK1, RIPK3, NLRP3, etc. In addition to exerting anticancer effects through the regulation of epigenetics, the sensitivity of tumor cells to chemotherapeutic agents could be enhanced synergistically by quercetin (Bądziul et al., 2014; Kedhari Sundaram et al., 2019; Zhai et al., 2021). Surprisingly, the experiments conducted by Kovacovicova et al. suggested that the combination of dasatinib and quercetin did not synergistically increase the antitumor efficacy of adriamycin or remove adriamycin-induced HCC senescent cells, and even dasatinib + quercetin alone shown acute pro-tumorigenic effects (Kovacovicova et al., 2018). Given the comprehensive and complex pharmacological treatment strategy for oncology patients with underlying diseases, the use of quercetin needs to be carefully considered. Besides, several studies have found that the efficacy of quercetin for inducing cellular autophagy is strongly correlated with the concentration of administration, with the advantage of lower concentrations being more pronounced (Kim et al., 2013).

However, the current research still has some objective limitations, and many issues affecting the development of quercetin as a drug remain to be solved. Firstly, the low bioavailability, poor stability and weak tumor-targeted biodistribution of quercetin extremely limit its application as an anti-tumor drug. To address these challenges, researchers developed some strategies to increase the bioavailability of quercetin. As a carrier for quercetin, chitosan helps quercetin release into the target site in a sustained and controlled state through various epithelial systems, thus enhancing cellular uptake (Rashedi et al., 2019; Elsayed et al., 2021). Lipid nanoparticles are also effective in enhancing the bioavailability of quercetin (Lou et al., 2016; Ren et al., 2017; Chen et al., 2020). Hence, the development of novel carriers and overlays for quercetin to enhance its bioavailability and targeted tumor effect is a critical research direction in the future. Secondly, most of the currently available studies on the pharmacological effects of quercetin in the non-apoptotic mode of induction of cancer cells are dominated by animal and cellular experiments, whereas large-sample, multicenter randomized controlled clinical trials are still needed to explore its true efficacy on tumors, including side effects. Thirdly, considering that the appropriate transfer of drug doses from animal models to humans is important in the development of new drugs, we recommend that additional investigations must be conducted to determine the appropriate and most effective doses for human use. Last but not least, the effects of quercetin on cell death mechanisms such as mitotic catastrophe, ferroptosis, necroptosis, and pyroptosis need to be fully investigated in terms of network pharmacology and genomics.

## Abbreviations

ATG, Autophagy protein; BCL-2, B-cell leukemia/lymphoma-2; CDK, Cycle protein-dependent kinase; EMT, Epithelial-mesenchymal transition; GSDMD, Gasdermin D; GSDMs, Gasdermin family; GC, Gastric cancer; GCTB, Giant cell tumor of bone; GSH, Glutathione; HCC, Hepatocellular carcinoma; HDAC, Histone deacetylase; IPF, Idiopathic pulmonary fibrosis; IFN-γ, Interferon-gamma; IL-1β, Interleukin-1beta; IL-2, Interleukin-2; IL-10, Interleukin-10; IL-18, Interleukin-18; IR, Ionizing radiation; JAK2, Janus kinase 2; MDA, Malondialdehyde; mTOR, Mammalian target of rapamycin protein; MLKL, Mixed lineage kinase domain-like; NF-κB, Nuclear factor kappa-B; NLRP3, NLR family pyrin structural domain; NMA, nuclear morphometric analysis; OC, Ovarian cancer; PI3K, Phosphatidylinositol-3-kinase; PCD, Programmed cell death; Akt, Protein kinase B; ROS, Reactive oxygen species; RIPK1, Receptor-interacting serine-threonine kinase 1; SASP, Senescence-associated secretory phenotype; STAT1, Signal transducer and activator of transcription-1; STAT3, Signal transducer and activator of transcription 3; TFEB, Transcription factor EB; TNF, Tumor necrosis factor.
